# Nonylphenol Induces Apoptosis through ROS/JNK Signaling in a Spermatogonia Cell Line

**DOI:** 10.3390/ijms22010307

**Published:** 2020-12-30

**Authors:** Hyun-Jung Park, Ran Lee, Hyunjin Yoo, Kwonho Hong, Hyuk Song

**Affiliations:** Department of Stem Cell and Regenerative Biotechnology, Konkuk University, 1 Hwayang-dong, Gwangjin-gu, Seoul 05029, Korea; sunrisep@konkuk.ac.kr (H.-J.P.); lanyi88@hanmail.net (R.L.); hyunjinyoo7@gmail.com (H.Y.); hongk@konkuk.ac.kr (K.H.)

**Keywords:** Nonylphenol, GC-1 spg cells, reproductive toxicity, Apoptosis, reactive oxygen species

## Abstract

Nonylphenol (NP) is an endocrine-disruptor chemical that negatively affects reproductive health. Testes exposure to NP results in testicular structure disruption and a reduction in testicular size and testosterone levels. However, the effects of NP on spermatogonia in testes have not been fully elucidated. In this study, the molecular mechanisms of NP in GC-1 spermatogonia (spg) cells were investigated. We found that cell viability significantly decreased and apoptosis increased in a dose-dependent manner when GC-1 spg cells were exposed to NP. Furthermore, the expression levels of the pro-apoptotic proteins increased, whereas anti-apoptosis markers decreased in NP-exposed GC-1 spg cells. We also found that NP increased reactive oxygen species (ROS) generation, suggesting that ROS-induced activation of the MAPK signaling pathway is the molecular mechanism of NP-induced apoptosis in GC-1 spg cells. Thus, NP could induce c-Jun phosphorylation; dose-dependent expression of JNK, MKK4, p53, and p38; and the subsequent inhibition of ERK1/2 and MEK1/2 phosphorylation. The genes involved in apoptosis and JNK signaling were also upregulated in GC-1 spg cells treated with NP compared to those in the controls. Our findings suggest that NP induces apoptosis through ROS/JNK signaling in GC-1 spg cells.

## 1. Introduction

Nonylphenol (NP) is a xenobiotic used in chemical manufacturing that can accumulate in the environment. Its accumulation in water, soil, and air allows NP to enter the food chain [1]. NP is produced from the environmental degradation of nonylphenol ethoxylates, which are toxic nonylphenol metabolites [1,2]. In addition, NP has been detected in human biological fluids, including plasma, breast milk, and urine [3,4,5]. NP is a well-known endocrine disruptor chemical (EDC) that causes hormone imbalance; upon accumulation in the body, it can result in reproductive and developmental disorders in humans [6,7]. The toxic effects of NP on the reproductive systems and gonads of mammals, fish, and reptiles have been studied [8,9]. NP can induce oxidative stress in the epididymal sperm of rats, reduce testis size and testosterone levels, and disrupt testicular structure and spermatogenesis [9,10,11]. In Sertoli cell cultures, NP treatment induced apoptosis by inhibiting the Ca^2+^ pump in the endoplasmic reticulum and increased oxidative stress [12]. NP also promotes apoptosis and autophagy in rat ovarian granulosa cells (GCs) [13].

Spermatogonia (spg) are undifferentiated male germ cells that undergo spermatogenesis to produce sperm in the testes. There is a growing concern that exposure to EDCs such as NP and bisphenol A (BPA) can affect fertility and sexual maturation. Indeed, damage by EDCs exposure at the early stage of germ-cell development, such as in the case of spermatogonia, can cause male infertility [14,15]. Although we previously reported that NP exposure in neonatal testes resulted in complete germ cell depletion and spermatogenic failure [16], the study did not address the specific mechanism of germ cell death. Therefore, the molecular mechanism of NP toxicity on germ cell survival and development is important for understanding the causes of male infertility and to develop novel treatment strategies. In addition, in vitro tests for assessing EDC toxicity, including that of NP, can be used to replace experiments that require animal euthanization. Furthermore, it is difficult to study the cellular and molecular mechanism of EDCs in specific cells in vivo because testes development and function is a complex process.

Reactive oxygen species (ROS) are essential for the regulation of physiological activity including cell proliferation, differentiation, migration, cell cycle progression, and cell death [17]. Several environmental endocrine-disruptors can increase oxidative stress and ROS production within cells and tissues, which can eventually lead to the activation of cell-death processes such as apoptosis [18,19]. Diverse signaling pathways are activated by ROS, including the mitogen-activated protein kinase (MAPK), phosphoinositide 3-kinase/Akt, nuclear factor (erythroid-derived 2)-like 2/Kelch like-ECH-associated protein 1, nuclear factor-κB, and the tumor suppressor p53 pathways [20,21]. However, the signaling pathways initiated by ROS can differ depending on the stimulus and cell type. Several studies have reported that EDCs induce ROS-mediated apoptosis. For example, BPA is a widely used industrial compound that can trigger apoptosis via ROS signaling in human neuroblastoma cells [22], mouse testes [23], and rat brains [24]. Octylphenol, an alkylphenol, is another EDC that is similar to NP and can also induce apoptosis through ROS generation in mouse testicular cells, such as Leydig and Sertoli cells [25,26]. Exposure to 2,3,7,8-tetrachlorodibenzo-p-dioxin is reported to induce apoptosis through oxidative damage and mitochondrial dysfunction in human trophoblast-like JAR cells and mouse Sertoli cells [27,28].

Previous studies have shown that apoptotic cell death can occur in reproductive tissues and cells exposed to NP. For example, in Sertoli cells derived from 18–20-day-old male rats, NP treatment induced apoptosis via the ROS- mediated AMPK/Akt-mTOR and JNK pathways [29]. NP also induced apoptotic cell death in mouse TM4 Sertoli cells through the transient activation of ERK signaling [30]. In rat ovarian GCs, NP induced oxidative stress and apoptosis via the Akt/AMPK/mTOR pathway [13]. Additionally, an in vivo study showed that BPA and NP induced rat germ cell apoptosis mediated by the activation of p38 MAPK and ADAM17 [31]. Although these and other studies have reported the toxic effects of NP in reproductive organs [8,16,30], the molecular mechanisms of NP toxicity in GC-1 spg cells have not been characterized in detail. Therefore, we investigated the molecular mechanisms underlying NP-mediated toxicity in GC-1 spg cells.

## 2. Results

### 2.1. NP Reduces Cell Viability in the GC-1 spg Cell Line

**Figure 1:**
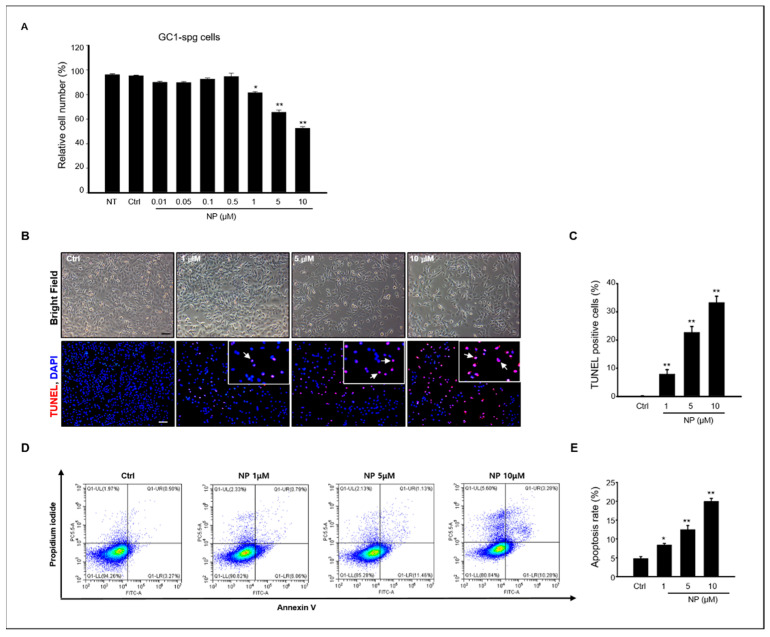
Effects of NP on viability and apoptosis rate of GC-1 spg cells. (**A**) GC-1 spermatogonia (spg) cell viability was measured using an MTT assay after a 24-h treatment with 0–10 μM NP and untreated (NT). The difference between 0.01–10 μM NP treatment groups was statistically significant compared to the controls. All data are presented as mean ± SD from three independent experiments (*n* = 3, * *p* < 0.05 and ** *p* < 0). (**B**) Detection of in situ DNA breaks using the TUNEL assay. TUNEL-positive nuclei (arrow) increase in a dose-dependent manner in NP-treated GC-1 spg cells. Scale bar = 100 μm. (**C**) The percentage of TUNEL-positive cells in each sample was determined and is expressed as the mean ± SD of three independent experiments. (*n* = 3, * *p* < 0.05 and ** *p* < 0.01 compared to the controls). Annexin V-FITC/PI staining was used to measure the apoptosis rate in GC-1 spg cells treated with 0, 1, 5, and 10 μM NP. (**D**) Representative dot plots of FACS analysis. (**E**) Quantitative analysis of the data as a percentage of apoptotic cells. Data are presented as the mean ± SD from three independent experiments (*n* = 3, * *p* < 0.05 and ** *p* < 0.01 compared to the controls).

A previous study reported that NP induced germ cell apoptosis in organ-cultured neonatal testes [16]. Based on these previous results, we evaluated the effects of NP in vitro using the GC-1 spg cell line, which was derived from 10-day-old mouse testes. First, to determine the cytotoxic effect of NP on the proliferation of GC-1 spg cells, a 3-(4,5-dimethylthiazol-2-yl)-2,5-diphenyltetrazolium bromide (MTT) assay was used to measure cell viability, which significantly decreased when the cells were exposed to 1–10 μM NP dissolved in 0.1% dimethyl sulfoxide (DMSO) for 24 h compared to the control group treated with 0.1% DMSO alone (Figure 1A). Based on this, the MTT results showed that the LC_50_ was 5.7 μM NP, and concentrations of 1–10 μM NP were used for further experimentation (Figure 1A).

### 2.2. NP Induces Apoptotic Cell Death in GC-1 spg Cells

We examined apoptosis using terminal deoxynucleotidyl transferase dUTP nick end labeling (TUNEL) to evaluate the cell death mechanism in NP-treated GC-1 spg cells. As shown in Figure 1B, TUNEL-positive cells increased in GC-1 spg cells treated with NP compared to the control. Moreover, the percentage of TUNEL-positive cells in NP-treated samples increased in a dose-dependent manner (Figure 1B,C). To determine the percentage of early and late apoptotic cells in NP-treated cultures, conventional flow cytometry was conducted with Annexin V fluorescein isothiocyanate (FITC) and propidium iodide (PI) labeling. Early apoptotic cells were visualized with Annexin V-FITC+/PI- staining patterns, whereas late apoptotic cells exhibited an Annexin V-FITC+/PI+ staining pattern. Our results revealed that both early and late apoptotic cells distinctly increased after NP treatment when compared with untreated control cell cultures, with approximately 20% of the cells observed to be apoptotic after treatment with 10 μM NP. The rate of apoptosis increased in an NP dose-dependent manner (Figure 1D,E), further suggesting that NP reduced cell viability through apoptotic mechanisms.

### 2.3. NP Induces the Expression of Pro-Apoptotic Proteins in GC-1 spg Cells

**Figure 2:**
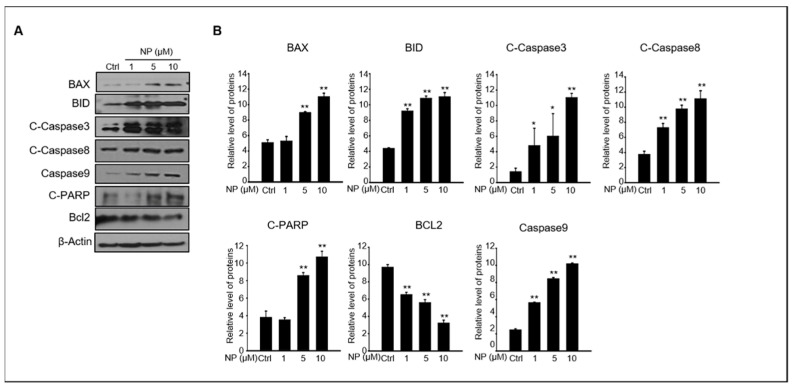
The effects of NP on pro-apoptotic protein expression in GC-1 spg cells. (**A**) The protein expression levels of BAX, BID, cleaved caspase-3 and caspase-8, cleaved-PARP, BCL2, caspase 9, and β-actin in GC-1 spg cells after treatment with 0, 1, 5, and 10 μM NP for 24 h. (**B**) Quantitative analysis of BAX, BID, cleaved caspase-3 and caspase-8, cleaved-PARP, BCL2, and caspase 9 protein expression levels. Graphs represent the relative density of each protein band normalized to that of β-actin. Data are presented as the mean ± SD of three independent experiments (*n* = 3, * *p* < 0.05 and ** *p* < 0.01 compared to the controls).

Next, we wanted to understand the mechanism of NP-induced apoptosis in GC-1 spg cells. The protein levels of key intrinsic and extrinsic apoptotic pathways such as BAX, BID, cleaved caspase-3, cleaved caspase-8, caspase-9, cleaved PARP, and BCL2 were normalized to β-actin protein levels to quantify the changes observed between GC-1 spg cells treated with 1–10 μM NP and control conditions (Figure 2A,B). We found that the levels of BAX, BID, cleaved caspase-3, cleaved caspase-8, caspase-9, and cleaved PARP were upregulated by NP treatment compared to that of the control. In contrast, the expression of BCL2 was downregulated in a dose-dependent manner in NP-treated cell cultures.

**Figure 3:**
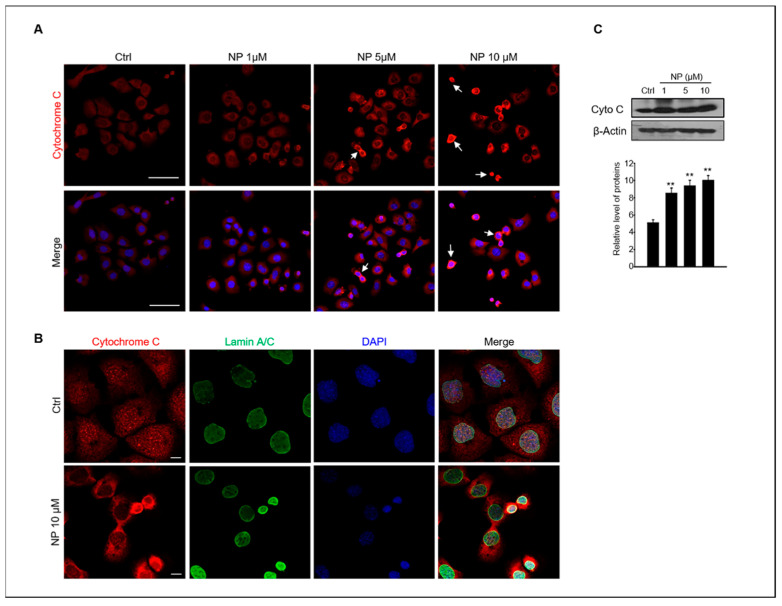
NP induces cytochrome c release in GC-1 spg cells. (**A**) GC-1 spg cells were treated with 0, 1, 5, and 10 μM NP for 24 h and then labeled with a cytochrome c (red, white arrow) antibody and co-labeled with DAPI to visualize cell nuclei with confocal microscopy. Scale bar, 50 μm. (**B**) labeled with a cytochrome c antibody and co-labeled with Lamin A/C to visualize the nuclear envelope. Scale bar, 10 μm. (**C**) Western blot of cytochrome c in GC-1 spg cells treated with 0, 1, 5, and 10 μM NP for 24 h. The density of cytochrome c bands was normalized to that of β-actin. Data are shown as the mean ± SD of three independent experiments (*n* = 3, ** *p* < 0.01 compared to the controls).

Stress-induced apoptosis can induce cytochrome c release from the mitochondria as well as result in caspase activation [31]. Therefore, we also examined whether NP could induce the release of cytochrome c in GC-1 spg cells. The cellular localization and protein expression of cytochrome c in GC-1 spg cells were examined using confocal immunofluorescence microscopy and Western blotting, respectively. The results showed strong cytochrome c immunofluorescence in GC-1 spg cells treated with 10 μM NP and a diffuse localization pattern in cells treated with 5–10 μM NP when compared with the untreated control. Indeed, cytochrome c was redistributed the region surrounding the LaminA/C + nucleus envelop in NP-treated cells (Figure 3A,B).

### 2.4. NP Induces ROS Production and Increases Mitochondrial Oxidative Stress in GC-1 spg Cells

**Figure 4:**
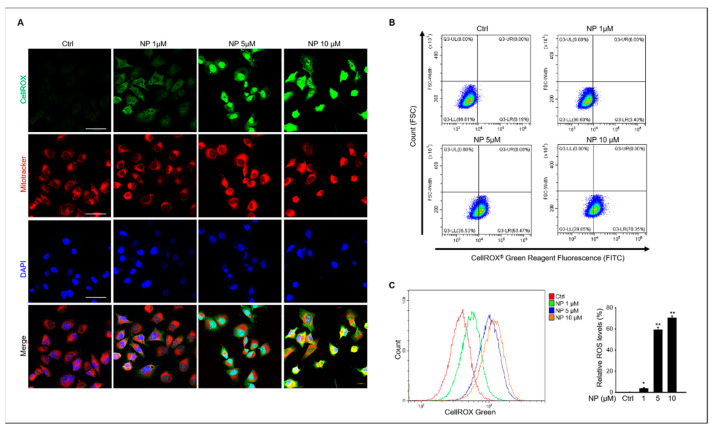
The effects of NP on mitochondrial reactive oxygen species production in GC-1 spg cells. (**A**) Treatment with 0, 1, 5, and 10 μM NP. GC-1 spg cells were double-stained with CellROX and MitoTracker Red CMXRos to measure oxidative-stress levels. The green signal indicates oxidative stress, whereas the red signal indicates changes in mitochondrial membrane potential. All images were obtained at the same magnification and the scale bar = 50 μm. (**B**) Flow cytometry plots for GC-1 spg cells stained with CellROX to measure oxidative stress levels. (**C**) Graphs represent the quantitative change of CellROX in NP-treated GC-1 spg cells. Data show the mean ± SD of three independent experiments (*n* = 3, * *p* < 0.05 and ** *p* < 0.01 compared to the controls).

Based on our results, we next tested for intracellular ROS generation in GC-1 spg cells after NP treatment. The results showed that 10 μM NP triggered ROS production in GC- 1 spg cells, which was indicated by an elevated CellROX^®^ green signal observed in NP-treated cells compared to that of the untreated control samples (Figure 4A). Additionally, mitochondrial ROS production was measured in NP-treated cells that also indicated increased mitochondrial oxidative stress via an enhanced red fluorescence signal in treated cells compared with the control (Figure 4A). ROS production was also measured using flow cytometry and CellROX^®^ labeling in GC-1 spg cells after NP treatment, which showed that 1–10 μM NP in the culture medium significantly increased ROS production in a dose-dependent manner (Figure 4B,C).

### 2.5. NP Induces Cell Death through ROS-Mediated JNK-Dependent Apoptosis

**Figure 5:**
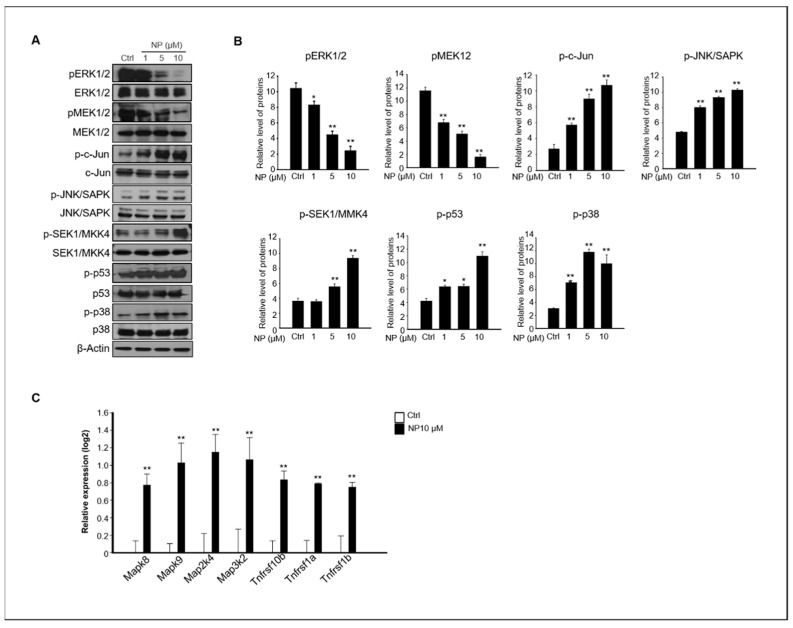
The effects of NP on MAPK activity. (**A**) The protein expression levels of pERK1/2, T-ERK1/2, pMEK1/2, p-c-Jun, p-JNK, p-MMK4, p-p53, p-p38, and β-actin in cells treated with 0, 1, 5, and 10 μM NP for 24 h. (**B**) The graph represents the densitometric analysis of each band normalized to that of each inactive protein band. (**C**) The gene expression levels of *Mapk8, Mapk9, Map2k4, Map3k2, Tnfrsf10b, Tnfrsf1a,* and *Tnfrsf1b* in GC-1 spg cells treated with 10 μM NP compared with untreated controls. Values represent the mean ± SD of three independent experiments (*n* = 3, * *p* < 0.05 and ** *p* < 0.01 compared to the controls).

The MAPK-mediated pathway plays an important role in apoptosis, and it has been reported that ROS are potent inducers of the MAPK pathway through JNK activation [20,32,33]. Our results showed that NP-treated GC-1 spg cells presented apoptosis-mediated accumulation of cellular ROS (Figure 4). Thus, we examined whether MAPK activation was involved in NP-induced GC-1 spg cell apoptosis. Treatment of GC-1 spg cells with NP resulted in the inhibition of ERK1/2 and MEK1/2 phosphorylation, but significantly increased the phosphorylation of c-Jun, JNK, MKK4, p53, and p38 expression in a dose-dependent manner compared to that observed in the untreated samples (Figure 5A,B). In addition, the gene expression levels of *Mapk8, Mapk9, Map2k4, Map3k2, Tnfrsf10b, Tnfrsf1a,* and *Tnfrsf1b*, which are related to apoptosis and JNK signaling, significantly increased in GC-1 spg cells treated with 10 μM NP compared with those in the control samples (Figure 5C).

## 3. Discussion

EDCs in the environment negatively affect vertebrate reproductive systems, including the development of reproductive organs, by interfering with hormonal systems [34,35]. Previous studies have demonstrated that NP exposure can induce apoptosis in various cell types, such as human epithelial intestinal cells [36], embryonic stem cells [37], thymocytes [38], and neurons [39]. Although NP induces apoptosis in cells and has been detected in the human body, its detailed mechanism of toxicity in the reproductive system has not been fully investigated.

In our previous study, we showed that NP can lead to germ cell depletion in pubertal testes but not Sertoli or Leydig cells [16]. Additionally, other studies reported that NP treatment can cause abnormal reproductive function in post-pubertal female rats, disrupt gonad development in both male and female rats [40], and decrease sperm counts, testes weight, and germ cell numbers [40,41]. Therefore, we dissected the molecular mechanisms of NP-induced apoptosis using the GC-1 spg cell line, which was derived from prepubertal testes. The present study showed that NP induced ROS-mediated apoptosis via the JNK signaling pathway in GC-1 spg cells.

Our results showed that GC-1 spg cell viability was reduced by approximately 40–50% after 10 μM NP treatment, and apoptosis rates increased by 20% with the same treatment compared with control conditions. There are major signaling pathways that can lead to cell death including apoptosis, autophagy, and necrosis. Duan et al. reported that NP induced autophagy, apoptosis, and necrosis in Sertoli cells [29], whereas our study focused on the ROS-mediated apoptotic pathway in GC-1 spg cells. It is possible that NP treatment induced GC-1 spg cell death through a mechanism other than apoptosis. Indeed, Sertoli cells that were exposed to NP showed programmed cell death via a ROS-mediated AMPK/Akt-mTOR and the JNK pathway. In our study, JNK also induced apoptosis in GC-1 spg cells treated with NP, consistent with another report that demonstrated that the JNK pathway is activated by increased ROS and induces autophagy in Sertoli cells [29].

Excess oxidative stress and ROS cause damage to DNA, proteins, lipids, and membranes, which can all lead to apoptotic cell death [19]. NP is toxic to both humans and animals [40,42]; the direct exposure to NP induces oxidative stress and apoptosis, previously reported in rat testicular Sertoli cells [8], ovarian GCs [13], and mouse oocytes [43] that are similar to those presented in this study. In addition to the reproductive system, NP increases ROS levels and induces oxidative damage in the liver, pancreas, and kidneys of rats [44,45,46]. GC-1 spg cells are type B spg cells that are considered to be a type of testicular germ cell, although they are not spermatogonial stem cells (SSCs). SSCs that are type A single (As) spg are located on the basement membrane of the seminiferous tubules. These cells can self-renew or produce the type A paired spg that undergo active mitosis, which further divide to produce type B spg cells that can then form primary spermatocytes that undergo meiosis [47]. Our previous study reported that NP downregulated the transcript levels of both undifferentiated and differentiated germ cell markers in neonatal mouse testes [16]. Therefore, we examined the effects of NP on apoptosis in type B spg cells, which is not a type of stem cell, in this study. Lei et al. reported that the cytotoxic effect of NP on SSCs occurs through the phosphatidylinositol-3-kinase/protein kinase B/mammalian target of rapamycin pathway. Indeed, the expression levels of SSC stemness maintenance markers, Nanog, Oct4, and Sox2, and the differentiation markers Nanos3, Stra8, Scp3, GFRα1, CD90, VASA, Nanos2, KIT, and PLZF, significantly decreased in SSCs treated with 10–30 μM NP compared with those in the controls. Moreover, the expression of Bad, cytochrome-c, and pro-Caspase 9 increased in NP-treated SSCs [48], which is consistent with our results. Therefore, although ROS are essential for the regulation of the normal physiological functions of the cell, such as proliferation and differentiation, and for maintaining normal immune responses [17,20], increased ROS levels can also negatively impact cell survival and development.

ROS-induced apoptosis has been reported in various cell systems. During apoptosis, there are two major programs that are downstream of the death signal: the caspase pathway and mitochondria and ER dysfunction, which are known as the extrinsic and intrinsic pathways, respectively [49]. In our study, apoptosis was induced by NP exposure in GC-1 spg cells that were involved in mitochondrial ROS-mediated death signaling. In particular, the expression levels of pro-apoptotic BCL-2 family proteins, such as BAX and BID, were increased by NP treatment and promoted caspase activation. The permeabilization of the mitochondrial outer membrane by Bax/Bak during apoptosis is essential for the intrinsic pathway during apoptotic cell death because when the mitochondrial membrane permeability increases, the release of cytochrome c into the cytosol activates caspase and leads to the apoptotic phenotype [50,51,52]. These reported apoptotic processes were similar to those observed in our study. Additionally, Liu et al. reported NP toxicity in mouse Sertoli TM4 cells, which showed that cell viability decreased due to induced apoptosis and was accompanied by altered *Bcl-2* family mRNA expression, as well as the activation of caspases-3, the release of Ca^2+^, and increased ROS generation. NP also increased the activation of the MAPK pathway and inhibited the Akt pathway in Sertoli TM4 cells [53].

JNK proteins belong to the MAP-kinase superfamily and were initially studied as UV-responsive protein kinases involved in the transactivation of c-Jun by phosphorylating its N-terminal Ser-63 and Ser-73 residues [54,55]. A number of studies have been conducted on the mechanism of ROS-meditated JNK activation and signaling in the control of diverse cellular functions, including cell differentiation, proliferation, and apoptosis [19]. JNK signaling is involved in both extrinsic pathways that initiate death receptors (TNF-α, TRAIL known as TNFSF10, and FAS-L) and intrinsic apoptotic pathways that are initiated by mitochondrial events [56]. Similar to the results presented in these previous reports, we found that the expression levels of *Tnfrsf10b*, *Tnfrsf1a*, and *Tnfrsf1b* were significantly increased and that mitochondrial dysfunction increased in GC-1 spg cells treated with 10 μM NP. In other words, both extrinsic and intrinsic apoptosis pathways are associated with JNK activation in response to NP exposure. In addition, apoptosis signaling through the JNK pathway indicates that these kinases function in a cell condition- and cell-type-specific manner to link these signals at different transmission points via both transcriptional and post-transcriptional mechanisms, which eventually converge on caspase activation [57].

MAPK signaling cascades include MAPK or ERK, MAPK kinase (MKK or MEK), and MAPK kinase kinase (MAPKKK or MEKK). MAPKKK/MEKK phosphorylates and activates its downstream protein kinase, MAPKK/MEK, which in turn activates MAPK [58]. There are multiple upstream components of the c-Jun N-terminal kinase (JNK/ MAPK) cascade, such as MAP3K1 (MEKK1), MAP3K2 (MEKK2), MAP2K7 (MKK7), and MAP2K4 (MKK4). In addition, the activation of JNK can lead to a multitude of downstream changes in phosphorylation, especially the JNK-potentiated transcriptional activity of c-Jun through the phosphorylation of serines 63 and 73, which modulate apoptosis [57,59]. Consistent with these previous studies, we also observed increased JNK, c-Jun, and MKK4 phosphorylation and *Mapk8* (*JNK1*), *Mapk9* (*JNK2*), *Map2k4* (*MKK4*), *Map3k2* (*MEKK2*), and *Jun* mRNA expression in NP-treated GC-1 spg cells.

It has been reported that MAPK8, MAPK9, and p38 (MAPK14) are substrates for MAP2K4; however, ERK1/2 is not phosphorylated by MAP2K4 even though ERK1/2 is a MAPK family protein with typical cascade signaling characteristics [60]. Phosphorylated p44 MAPK and p42 MAPK, which are known as ERK1 and ERK2, translocate into the nucleus from the cytoplasm to regulate the transcription factors and gene expression that promote cell growth, differentiation, or mitosis in cells. Although activation of ERK/MEK is generally associated with cell proliferation, some studies have shown that ERK mediates cell death. However, the molecular mechanisms of ERK-mediated cell death are still not fully understood [61]. Our results suggested that the suppression of MEK/ERK activation by NP probably led to the inhibition of cell growth and was linked to cell death. Moreover, NP stimulated p53 and p38 phosphorylation through the JNK pathway in GC-1 spg cells. There is evidence that JNK mediates p53 activation; studies have reported that JNK can phosphorylate p53 [59,62]. JNK activity requires MEKK, which phosphorylates MKK4/7 and JNK on residues 183 and 185; the activated JNK phosphorylates its substrates c-Jun, ELK1, and p53 [59,63,64]. Our results revealed increased JNK pathway activation and phosphorylated p53 and p38 in NP-treated GC-1 spg cells, suggesting a correlation between JNK and p38. The p38 MAPK is a member of the MAPK subfamily that is responsive to stress stimuli, including ultraviolet irradiation, heat shock, and osmotic shock; however, it is also involved in cell apoptosis, autophagy, and cell differentiation [65,66]. The phosphorylation and activation of p38 can not only mediate cell apoptosis [67,68] but also exert protective effects on cells [32,69]. Both JNK and p38 MAPK cascades mediate apoptotic processes, and several Bcl-2 family proteins are under the control of JNK and/or p38 MAPK cascades at transcriptional and/or post-transcriptional levels [70,71].

In conclusion, although the concentration of NP is higher than physiological NP levels observed in humans [72], NP exposure resulted in decreased GC-1 spg cell viability; increased the expression of apoptosis-related proteins, BAX, BID, cleaved caspase-3 and caspase 8, caspase 9, and cleaved PARP; and decreased Bcl-2 expression. NP treatment also led to the accumulation of ROS and increased oxidative stress and apoptosis mediated by abnormal mitochondrial membrane potential in GC-1 spg cells. In addition, NP activated the MAPK family-associated proteins, JNK, c-Jun, p38, and MKK4 and also suppressed ERK1/2 and MEK1/2 activity. The results from our study suggested that these molecular mechanisms are involved in the toxic effect of NP on spermatogonia cells in vitro.

## 4. Material and Methods

### 4.1. Cell Culture and Treatments

The mouse GC-1 spg (spermatogonia) cell line was purchased from the Korean Cell Line Bank (KCLB 21715, Seoul, South Korea) and cultured in Dulbecco’s modified Eagle’s medium supplemented with 10% fetal bovine serum and 1% penicillin-streptomycin in a humidified atmosphere of 5% CO_2_ at 37 °C. NP (Sigma-Aldrich St. Louis, MO, USA) was dissolved in DMSO to prepare a 1 M stock solution, which was diluted to the desired concentration using the cell culture medium prior to cell culture.

### 4.2. Cell Viability Determination Using MTT Assay

Cell viability was determined with an MTT assay using the EZ-Cytox Viability assay kit (Daeil Lab Services Co, Seoul, Korea, #EZ1000) and following the manufacturer’s instructions to measure the half-maximal inhibitory concentration (IC_50_) of NP in GC-1 spg cells. GC-1 spg cells were seeded in 96-well plates at a density of 5 × 10^3^/well in culture medium and incubated for 24 h at 37 °C. The culture medium was replaced with fresh medium containing different concentrations of NP (0.01–10 μM) and cultured for an additional 24 h. The assay reagent was added, and cultures were incubated for 60 min before the plates were read at 490 nm using a Sunrise^TM^ spectrophotometer (TECAN, Männedorf, Switzerland).

### 4.3. Apoptosis Assay

TUNEL assay was used to quantify DNA and chromatin morphogenic features. For TUNEL staining, an in situ TMR red cell death detection kit (Roche, Germany) was used following the manufacturer’s guidelines. Cells were cultured on glass slides for 24 h and then exposed to 0, 1, 5, or 10 µM NP for 24 h. The cells grown on coverslips were washed twice with phosphate-buffered saline (PBS; Sigma-Aldrich) and fixed with 4% paraformaldehyde in PBS for 15 min at 24 °C. Following the washes, the cells were incubated in a permeabilization solution (0.1% Triton X-100 in PBS) for 2 min on ice. The samples were incubated in 50 μL TUNEL reaction mixture (Roche, Mannheim, Germany) for 60 min at 37 °C in a humidified chamber in the dark. Samples were incubated with or without 1 µg/mL 6-diamidino-2-phenylindole (DAPI) in PBS for 5 min; coverslips were applied using the mounting solution (Dako, Carpinteria, CA, USA; S3025) and imaged using fluorescence microscopy (Nikon, Tokyo, Japan).

### 4.4. Flow Cytometry

Annexin V-FITC staining assay was conducted using the dead cell apoptosis kit (Thermo Fisher Scientific, Inc., Waltham, MA, USA), which uses Annexin V-FITC and PI, to measure apoptosis in GC-1 spg cells after NP treatment. The cells were seeded in six-well plates and treated with NP for 24 h. The cells were then collected after trypsinization and washed with PBS before being stained with Annexin V-FITC and PI for 15 min at 24 °C in the dark. Annexin V-positive cells were detected using flow cytometry (CytoFLEX, Beckman Coulter, Inc., Miami, FL, USA). ROS generation was determined using the CellROX Green flow cytometry assay kit (Thermo Fisher Scientific, San Jose, CA). Samples were stained according to the manufacturer‘s instructions, and the fluorescence intensity of CellROX Green was measured with a flow cytometer (CytoFLEX, Beckman Coulter, Inc., Miami, FL, USA).

### 4.5. Immunofluorescence Staining

ROS generation was assessed in GC-1 spg cells treated with 10 µM NP using CellROX Oxidative stress reagents. GC-1 spg cells were seeded onto 12-mm glass coverslips (BD Biosciences, Franklin Lakes, NJ) at a density of 1 × 10^5^ cells/coverslip and allowed to attach for 1 d prior to treatment with 10 µM NP for 24 h. The cells were then used for immunofluorescence staining with CellROX^®^ green (C10444, Life Technologies, Carlsbad, CA, USA) and MitoTracker^®^ red CMXRos (M7512, Life Technologies, Carlsbad, CA, USA). Twenty-four hours after NP treatment, CellROX green and DAPI were added to the cells and incubated for 30 min at 37 °C. The medium was then removed and the cells were washed three times with PBS. Cells were fixed in 4% formaldehyde for 15 min. For mitochondria staining, samples were incubated for 30 min with 100 nM MitoTracker^®^ red CMXRos at 37 °C and then fixed. All samples were mounted in mounting medium (Sigma-Aldrich), and images were obtained using confocal microscopy (Carl Zeiss, Oberkochen, Germany; LSM 800).

### 4.6. Isolation of RNA and Quantitative Real-Time PCR Analysis

Total RNA was extracted from GC-1 spg cells using a RNeasy Mini Kit (Qiagen, Hilden, Germany) with on-column DNase treatment (Qiagen). Complementary DNA (cDNA) was synthesized using SuperScript™ III Reverse Transcriptase (Invitrogen, Carlsbad, CA, USA) with an oligo(dT)30 primer following the manufacturer’s instructions. Template cDNA was mixed with iQ SYBR Green Supermix (170–8880; Bio-Rad Laboratories), and 1 pM of each primer was used for qPCR. Primers were designed using Primer3 (http://frodo.wi.mit.edu). The cycle threshold values were normalized against *GAPDH* gene expression. Denaturation and polymerase activation steps were performed at 94 °C for 1 min followed by 40 cycles at 94 °C for 10 s, 57 °C for 10 s, and 72 °C for 20 s. The primers used to detect mouse transcripts are listed in Table 1.

### 4.7. Western Blotting

Western blotting was conducted as described previously [32]. GC-1 spg cell lysates were prepared using RIPA lysis buffer (Thermo Scientific™, Rockford, IL, USA) and a protease inhibitor cocktail (Roche, Rotkreuz, Switzerland). Protein samples were separated in 4–12% gradient SDS-PAGE gels, and proteins were transferred to PVDF membranes using a transfer blotting system (Bio-Rad, Hercules, USA). Membranes were incubated in blocking buffer solution (TBS with 0.1% tween-20 (TBST) + 1% bovine serum albumin) for 1 h and then incubated with primary antibodies overnight at 4 °C. Membranes were washed with TBST for 30 min and then incubated for 1 h with secondary antibodies in TBST +1% BSA. All antibodies used in this study are listed in Table 2. Blots were visualized using Pierce ECL solution (Thermo Fisher Scientific, Rockford, IL, USA) and X-ray film. ImageJ software was used to quantify protein expression.

### 4.8. Statistical Analysis

All results are expressed as mean ± standard error values from at least three independent experiments and were evaluated using one-way analysis of variance. All statistical analyses were conducted using the SPSS statistical package, version 15.0 for Windows (IBM Corp, Somers, NY, USA). Values of * *p* < 0.05 and ** *p* < 0.01 were considered statistically significant.

## Figures and Tables

**Table 1:** Primers used for reverse transcription-polymerase chain reaction.

Gene	Forward Primer	Reverse Primer
*Mapk8*	5′-GAACAGGATTGAGTAGCGGC-3′	5′-TCATGATGGCAAGCAATTAGTC-3′
*Mapk9*	5′-CTGGAGCCCAAGGAATTGT-3′	5′-GCGTTTGGTTCTGAAAAGGA-3
*Map2k4*	5′-TAAACCGTGCTGTCGATTTG-3	5′-TCGGACCTCCAGCTTCTG-3
*Map3k2*	5′-GGGCCTCATCTTAACCCTTC-3′	5′-AGTCATCCGCGTGAAACAG-3′
*Tnfrsf10b*	5′-GCCTTGCAGAGAGGGTATTG-3′	5′-CATCGACACACCGTATTTGTG-3
*Tnfrsf1a*	5′-ACCAGTTCCAACGCTACCTG-3′	5′-TGCATGGCAGTTACACACG-3′
*Tnfrsf1b*	5′-GACACCCTACAAACCGGAAC-3′	5′-GCCAGGAGGACACTTAGCAC-3′
*Gapdh*	5′-GTCGGTGTGAACGGATTTG-3′	5′-CTTGCCGTGGGTAGAGTCAT-3′

**Table 2:** List of antibodies used for immunostaining and immunoblotting.

1° Antibody	Company	Catalogue Number	Dilution
BAX	Cell Signaling Technology	#5023	1:1500
BID	Cell Signaling Technology	#2003	1:1500
Cleaved-Caspase 3	Cell Signaling Technology	#9664	1:1500
Cleaved-Caspase 8	Cell Signaling Technology	#8592	1:1500
Caspase 9	Cell Signaling Technology	#9504	1:1500
Cleaved-PARP	Cell Signaling Technology	#5625	1:1500
BCL2	Cell Signaling Technology	#3498	1:1500
P-p44/42 MAPK	Cell Signaling Technology	#4370	1:2000
P44/42 MAPK	Cell Signaling Technology	#9102	1:2000
pMEK1/2	Cell Signaling Technology	#9154	1:2000
MEK1/2	Cell Signaling Technology	#9122	1:2000
p-c-Jun	Cell Signaling Technology	#3270	1:1500
c-Jun	Cell Signaling Technology	#9165	1:1500
p-JNK/SAPK	Cell Signaling Technology	#9255	1:1500
JNK	Cell Signaling Technology	#9252	1:1500
p-SEK1/MKK4	Cell Signaling Technology	#9156	1:1500
MKK4	Cell Signaling Technology	#9152	1:1500
p-p53	Cell Signaling Technology	#12571	1:1000
p53	Cell Signaling Technology	#2524	1:1000
p-p38	Santa Cruz Biotech	SC-166182	1:1000
p38	Cell Signaling Technology	#9212	1:1000
Cytochrome c	Abcam	Ab133504	1:1000
β-Actin	Santa Cruz Biotech	SC-47778	1:1000

## Abbreviations

Nonylphenol (NP); endocrine-disruptor chemical (EDC); reactive oxygen species (ROS); mitogen-activated protein kinase (MAPK); spermatogonia (spg); terminal deoxynucleotide transferase-mediated deoxy-UTP nick end labeling (TUNEL); Annexin V-fluorescein isothiocyanate (FITC); propidium iodide (PI); spermatogonial stem cell (SSC), bisphenol A (BPA).
